# Long noncoding RNA SNHG20 regulates cell migration, invasion, and proliferation via the microRNA-19b-3p/RAB14 axis in oral squamous cell carcinoma

**DOI:** 10.1080/21655979.2021.1950278

**Published:** 2021-07-20

**Authors:** Xiaomi Zhu, Hanzhong Zhang, Juan Xu

**Affiliations:** a Department of Stomatology, Hubei Hospital of Traditional Chinese Medicine, Wuhan, Hubei, PRR China; b Department of Stomatology, The Central Hospital of Wuhan, Tongji Medical College, Huazhong University of Science and Technology, PRR China; c Department of Stomatology, People Hospital of Lishui, PRR China

**Keywords:** Lncrna snhg20, miR-19b-3p, RAB14, OSCC, proliferation

## Abstract

Oral squamous cell carcinoma (OSCC) is one of the most common digestive tumors, which has high mortality rate. Long non-coding RNAs (lncRNA) and MicroRNAs (miRNAs) are associated with the cell cycle and differentiation during the occurrence and development of malignant tumors. This research aimed to investigate the effects of lncRNA SNHG20 on the progress of oral squamous cell carcinoma (OSCC) cells. Ninety pairs of tumor tissues and paracancerous tissues were collected from patients with OSCC and the CAL27 and SCC25 OSCC cells were selected for the following experiments. RT-qPCR was used for detecting the expression of SNHG20, miR-19b-3p, and RAB14. Western blotting was used to detect the protein levels of RAB14. MTT assay was employed to assess cell proliferation. Transwell assay was used to determine the cell migration and invasion abilities. Furthermore, luciferase reporter and RNA pull-down assays were used to verify the binding of SNHG20/RAB14 to miR-19b-3p. Then, the function of the SNHG20/miR-19b-3p/RAB14 axis in OSCC was explored. The results indicated that lncRNA SNHG20 was upregulated in the tissues. Furthermore, bioinformatic analysis showed that both SNHG20 and RAB14 could bind to miR-19b-3p. RAB14 was upregulated, and miR-19b-3p was downregulated in the tissues. The knockdown of SNHG20 inhibited cell proliferation, migration, and invasion. Contrarily, the knockdown of miR-19b-3p reversed the effects of si-SNHG20 on cell proliferation, migration, and invasion, and the overexpression of RAB14 reversed the effects of miR-19b-3p mimic on the cell biological functions. LncRNA SNHG20 affects cell proliferation, migration, and invasion via the miR-19b-3p/RAB14 axis in OSCC.

## Graphical abstract

**Figure:**
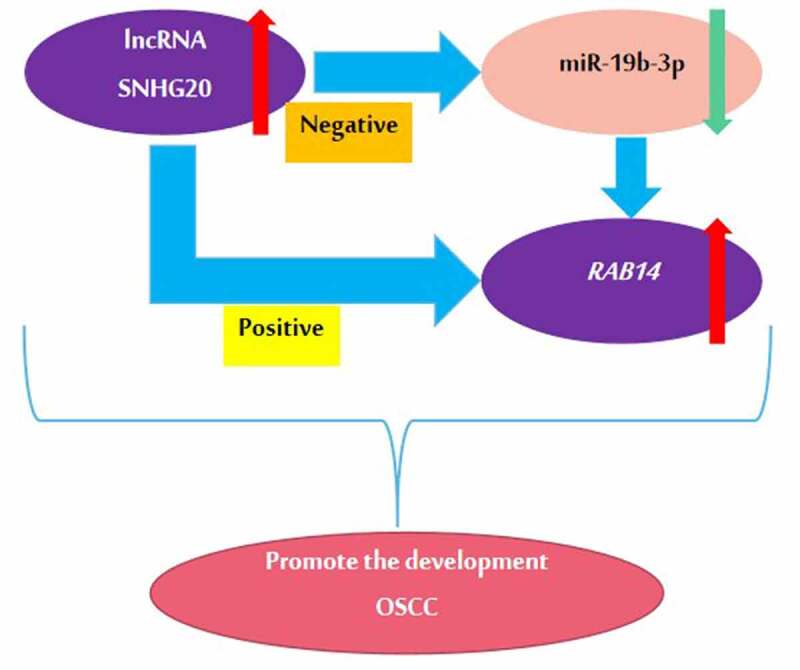

## Introduction

Oral cancer is one of the most common digestive tumors and is ranked as the thirteenth most common cancer in the world [1]. It was reported that the exposure to tobacco smoke, chemicals, radiation, and air pollution could induce the occurrence of oral cancer [2,3]. Oral squamous cell carcinoma (OSCC) makes up the majority of the oral cancer cases [4,5]. The generally used OSCC treatment approaches are chemotherapy and targeted therapy [6]. However, due to late diagnosis and drug resistance, the 5-year survival rate of patients remains below 17% [7]. Therefore, it is critical to develop novel anti-OSCC therapies to improve the life quality and survival rate of patients.

Long noncoding RNAs (lncRNAs) are significant regulators in different cellular processes [8]. Furthermore, most lncRNAs are transcribed by RNA polymerase II; therefore, they are similar to messenger RNAs (mRNAs), involving a 5'-7-methylguanosine cap and a 3' poly(A) tail. However, lncRNAs lack a coding capacity [9]. Meanwhile, lncRNAs are known as gene regulators that participate in different molecular events, such as chromatin modifications, chromatin structure, DNA methylation, genome organization, and interactions with transcription factors, at various levels, to influence the transcription of chromatin-related proteins and gene expression [10]. LncRNA small nucleolar RNA host gene 20 (GenBank Accession ID NR_027058.1), which belongs to the SNHG lncRNA family, was first reported in hepatocellular carcinoma (HCC) [11]. A previous study showed that the upregulation of lncRNA SNHG20 in OSCC could promote cell proliferation and migration [12]. However, the specific mechanism of lncRNA SNHG20 in OSCC remains unclear.

Rab protein, as a small GTP binding protein, circulates between the active GTP binding state and the inactive GDP binding state [13]. Rab protein is a molecular switch for intracellular and extracellular transport in all eukaryotes . RAB14 is a member of Rab GTPase family. RAB14 is involved in autophagy, lysosome activity, cell migration and membrane transport between Golgi and endosomes under normal physiological conditions [14]. In various tumor cells, RAB14 affects the development of tumor by regulating the proliferation, migration and invasion of tumor cells. Previous study confirmed that the mRNA expressions levels of RAB14 in gastric cancer was significantly higher than that in healthy gastric mucosa and the abnormal expression of RAB14 in gastric cancer promotes the malignant development of tumor by activating Akt signaling pathway [15]. In kidney cancer cells, Rab14 expression is significantly up-regulated, which promotes the proliferation, invasion, and metastasis of tumor cells and enhances the chemotherapy resistance of tumor cells [16]. In addition, the increased expression of RAB14 in ovarian cancer promotes tumor cell proliferation and invasion through Wnt signaling pathway, which is associated with poor prognosis [17]. However, the reports about RAB14 on the progress of OSSC is still limited.

Therefore, this research aimed to elucidate the specific mechanism of lncRNA SNHG20 during the progress of OSCC. We hypothesized that LncRNA SNHG20 affects cell proliferation, migration, and invasion via the miR-19b-3p/RAB14 axis.

## Materials and methods

### Patients and specimens

A total number of 90 patients with OSCC were recruited from the People Hospital of Lishui between October 2017 and October 2019. In brief, the para-carcinoma tissues that did not contain obvious cancer cells were collected 5–10 cm away from the border of the tumor. Subsequently, the tumor and the para-carcinoma tissues were fixed in 10% formalin. Additionally, an informed consent was signed by each patient. This study has been approved by the Ethics Committee of the People Hospital of Lishui.

### Cell lines

The NHOK, TSCCA, SCC15, SCC25, and CAL27 cell lines were collected from the Cell Bank of Chinese Academy of Sciences. All cell lines were kept in RPMI-1640 medium (Thermo Fisher Scientific, MA USA) containing 10% FBS (Biosera, MO, USA) and 100 U/mL streptomycin/penicillin (Thermo Fisher Scientific, MA USA) in a humid atmosphere containing 5% CO_2_ at 37°C.

### Cell culture and transfection

SiRNA targeting SNHG20 (si-SNHG20), miR-19b-3p inhibitor, miR-19b-3p mimic, overexpressed RAB14 (OE-RAB14), si-NC, inhibitor NC, mimic NC, and OE-NC were obtained from GenePharma (China, Soochow). Subsequently, the cells were inoculated on a 6-well plate and cultured in RPMI-1640 containing 5% CO_2_ at 37°C until the cells achieved 60% confluence. In brief, the plasmids were transfected into cells (1 × 10^5^ cells/well) using Lipofectamine^TM^ 2000 Transfection Reagent (Thermo Fisher Scientific, MA USA) based on the manufacturer’s instructions.

### MTT assay

Cells in the logarithmic growth phase were seeded on a 96-well plate. After incubation for 0 h, 24 h, 48 h, and 72 h, cells were treated with MTT in fresh medium for 4 h. Dimethyl sulfoxide was added to dissolve the formazan product. Furthermore, the optical absorbance was measured at 490 nm using a microplate reader.

### Luciferase reporter assay

The luciferase reporter assay was performed according to previous study [18]. The gene fragments containing the SNHG20/RAB14 binding site (HOTAIR-wild type (WT)) and the mutated SNHG20/RAB14 binding site (HOTAIR-MUT) were synthesized and cloned into the fluorescent vector psiCHECK2 to construct the luciferase reporter gene. Then, cells were co-transfected with mimic NC and miR-19b-3p mimic, respectively. According to the dual luciferase reporter gene test kit requirements, the luciferase activity of renilla and firefly was detected.

### RNA pull-down assay

The RNA pull-down assay was performed as described by Tang et al. [19]. CAL27 and SCC25 cells were lysed and incubated with biotinylated miR-19b-3p or biotinylated NC. The final concentration of each biotinylated miRNA was 20 nM. Two days later, the cell lysates were collected and then cultured with M-280 Streptavidin magnetic beads for 3 h. Then, they were washed three times. In the qRT-PCR analysis, TRIzol was employed to purify the bound RNA.

### RNA isolation and real-time quantitative PCR

We used TRIzol to collect the total RNA from tissues and cells based on the manufacturer’s instructions. The RNA concentration was analyzed using NanoDrop 2000 (Thermo, America). The cDNAs were synthesized by using the PrimeScript RT reagent kit transcriptase, Random 6mers, RNase inhibitor, Oligo dT primer, a dNTP mixture, and reaction buffer. Quantitative real-time PCR (qRT-PCR) was performed using an SYBR Premix EX Taq™ kit (TAKARA, Tokyo, Japan). The cycle conditions used were: 95°C for 30 s, followed by 95°C for 5 s and 40 cycles at 60°C for 30 s each. We used the 2^−ΔΔCt^ method to analyze the data [20]. *GAPDH* or *U6* was selected as the internal reference gene.

The primers used in qRT-PCR were the following:

LncRNA SNHG20: forward, 5'-ATGGCTATAAATAGATACACGC-3'; reverse, 5'-GGTACAAACAGGGAGGGA-3'; miR-19b-3p: forward, 5'-ACACTCCAGCTGGGTGTGCAAATCCATGCAA-3',

reverse: 5'-CTCAACTGGTGTCGTGGAGTCGGCAATTCAGTTGAGTCAGTTT-3';

RAB14: forward, 5'-TATGGCTGATTGTCCTCACACA-3';

reverse: 5'-CTGTCCTGCCGTATCCCAAAT-3';

GAPDH forward: 5'-TGTGGGCATCAATGGATTTGG-3';

reverse: 5'-ACACCATGTATTCCGGGTCAAT-3'; and

U6: forward, 5'-AAAGCAAATCATCGGACGACC-3';

reverse: 5'-GTACAACACATTGTTTCCTCGGA-3'.

### Transwell assays

The Transwell method was employed to determine the growth and metastasis abilities of the OSCC cells described as a previous study [21]. At first, cell culture Transwell inserts (8 mm pore size; Falcon; BD Biosciences) have been placed into 48-well plates to generate upper and lower chambers. Furthermore, cells were seeded on the upper chamber with a Matrigel-coated membrane, for the migration assays, or in chambers not coated with Matrigel, for the invasion assays, while the lower wells were filled with culture media containing serum. After 24 h of culture, we used a counting chamber to count the number of migrating and invading cells.

### Western blot

The western blot was conducted according to a previous research [22]. Protein was collected from SCC25 and CAL27 cells using RIPA buffer (Sigma-Aldrich, MO, USA) and the concentration of total protein was determined using a BCA kit (Sigma-Aldrich, MO, USA). Additionally, the proteins (20 µg/lane) were isolated using a 15% SDS-PAGE gel, and then transferred to PVDF membranes (Bio-rad, California, USA), which were blocked with 5% skimmed milk for 2 h before the blotting procedure. Cells were incubated with the primary anti-RAB14 antibody overnight at 4°C. On the next day, the membranes were treated with the secondary antibodies. Subsequently, the membrane was stained using an ECL western blotting kit. At the same time, we used GAPDH as a loading control. The results were analyzed using Image J.

## Statistical methods

Data were examined using SPSS 21.0. All experiments were repeated three times and all data were presented as the mean ± standard deviation (X ± s). Furthermore, comparisons between two groups were analyzed using a t-test. Multiple-group comparisons were investigated using one-way ANOVA. A P < 0.05 was defined as a meaningful statistical difference.

## Results

The aim of this study was to explore the specific mechanism of lncRNA SNHG20 during the progress of OSCC. Through bioinformatic analysis, we found that LncRNA SNHG20 regulated OSCC cell functions by sponging miR-19b-3p and RAB14 is the target gene of miR-19b-3p in OSCC via. We hypothesized that LncRNA SNHG20 affects cell proliferation, migration, and invasion via the miR-19b-3p/RAB14 axis

### LncRNA SNHG20 was upregulated in OSCC tissues and SCC25 and CAL27 cells

**Figure 1.**
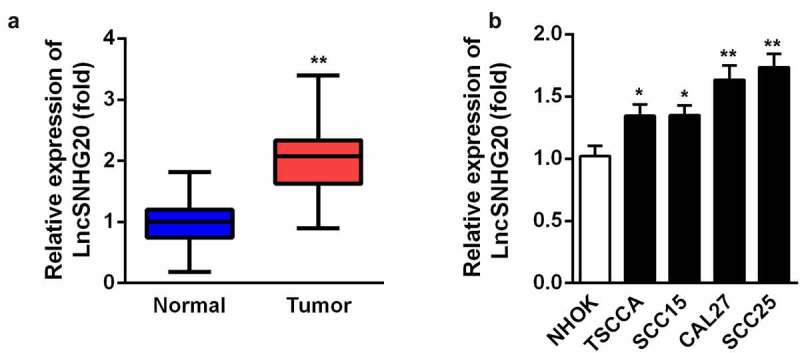
LncRNA SNHG20 was overexpressed in OSCC tissues and CAL27 and SCC25 cells

Firstly, we determined the expression of lncRNA SNHG20 in the OSCC samples by using RT-qPCR. Our data suggested that, compared with the adjacent non-tumor tissues, SNHG20 was dramatically upregulated in the OSCC tissues (Figure 1a). Then, we examined the level of lncRNA SNHG20 in OSCC cell lines (TSCCA, SCC15, SCC25, and CAL27) and used NHOK cells as control cells. We found that the expression of lncRNA SNHG20 was increased in TSCCA, SCC15, SCC25, and CAL27 cells; moreover, it was the highest in SCC25 and CAL27 cells (Figure 1b). Therefore, we used SCC25 and CAL27 cells in the following experiments.

RT-qPCR analysis of lncRNA SNHG20 in OSCC tissues (A) and cells (B). Data are presented as mean ± SD. * p < 0.05; ** p < 0.01.

### The knockdown of lncRNA SNHG20 inhibited the proliferation, migration, and invasion of SCC25 and CAL27 cells

**Figure 2.**
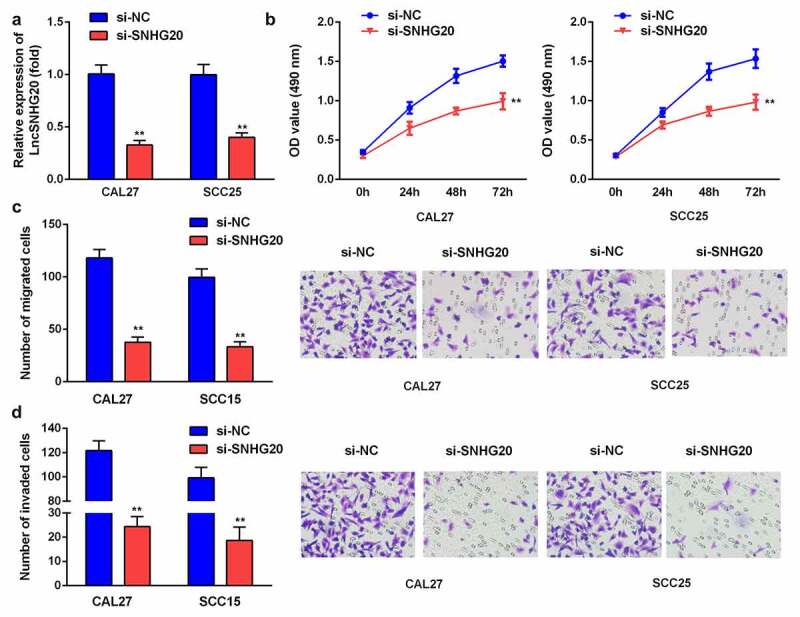
The knockdown of lncRNA SNHG20 decreased the proliferation, migration, and invasion of SCC25 and CAL27 cells

To explore the impact of lncRNA SNHG20 on SCC25 and CAL27 cells, we knocked down the lncRNA SNHG20 using siRNA. Compared with the si-NC group, the expression of SNHG20 was remarkably downregulated in the si-SNHG20 group (Figure 2a). In addition, compared with the si-NC group, the proliferation of SCC25 and CAL27 cells in the si-SNHG20 group was significantly decreased (Figure 2b). Similarly, the knockdown of lncRNA SNHG20 dramatically downregulated the growth and metastasis of SCC25 and CAL27 cells (Figure 2c-d).

(A) RT-qPCR analysis of lncRNA SNHG20 in OSCC cells transfected with si-SNHG20 and si-NC. (B) An MTT assay was used to explore the effect of si-SNHG20 on the proliferation of CAL27 and SCC25 cell lines. The number of migratory (C) and invasive (D) cells was determined using Transwell assays. Data are presented as mean ± SD. ** p < 0.01.

### LncRNA SNHG20 regulated OSCC cell functions by sponging miR-19b-3p

**Figure 3.**
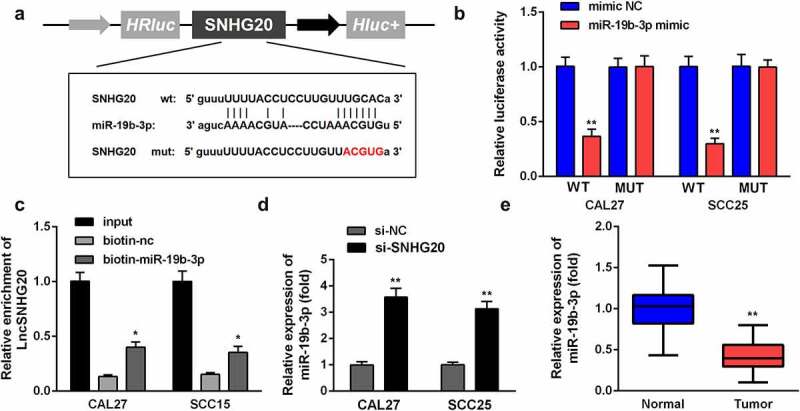
LncRNA SNHG20 regulates the OSCC cell functions by sponging miR-19b-3p

We predicted the targets of lncRNA CRNDE and searched miR-19b-3p for further analysis. The targeting sites of LncRNA SNHG20 and miR-19b-3p were estimated by using the online starBase software (Figure 3a). The luciferase reporter data showed that miR-19b-3p mimic sharply inhibited the luciferase activity in cells transfected with WT LncRNA SNHG20, rather than with Mut-LncRNA SNHG20, in SCC25 and CAL27 cells (Figure 3b). In addition, the luciferase reporter results were confirmed by using an lncRNA SNHG20 probe pull-down assay (Figure 3c). Moreover, we found that the knockdown of lncRNA SNHG20 can promote miR-19b-3p expression (Figure 3d). The RT-qPCR results showed that miR-19b-3p was dramatically downregulated in OSCC tissues, compared to the adjacent non-tumor tissues (Figure 3e).

(A) The predicted lncRNA SNHG20 binding site in the miR-19b-3p 3'-UTR. (B) The relative luciferase activity of cells after co-transfection with wild type (WT) or mutant (Mut) lncRNA SNHG20 3'-UTR reporter genes and miR-19b-3p mimics. (C) The RNA pull-down assay further confirmed that SNHG20 could bind to miR-19b-3p. (D) RT-qPCR analysis was used to explore the effect of si-SNHG20 on the miR-19b-3p level of CAL27 and SCC25 cells. (E) RT-qPCR analysis of miR-19b-3p in OSCC tissues. Data are presented as mean ± SD. * p < 0.05; ** p < 0.01.

### Knockdown of miR-19b-3p reversed the effects of si-SNHG20 on the proliferation, migration, and invasion of SCC25 and CAL27 cells

**Figure 4.**
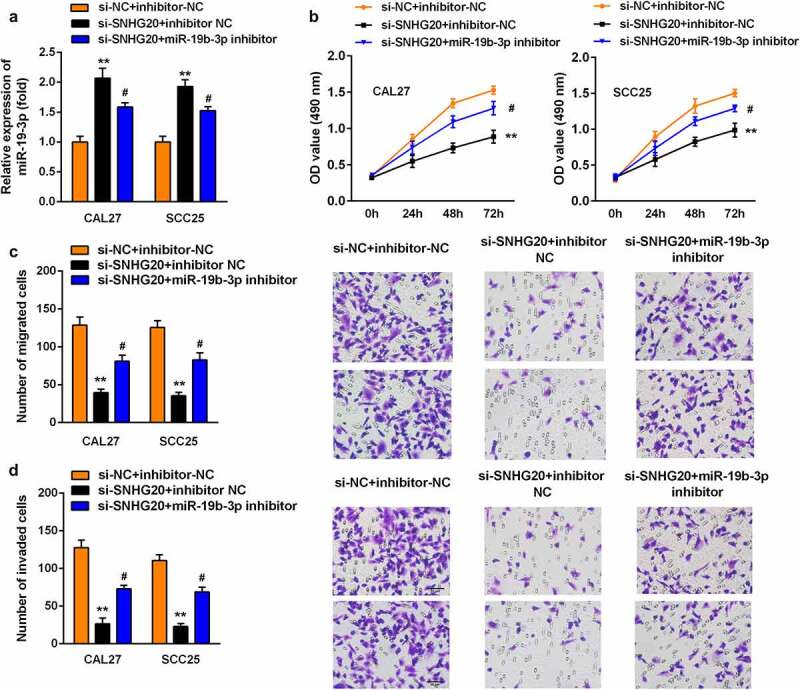
The knockdown of miR-19b-3p reversed the effect of si-SNHG20 on the proliferation, migration, and invasion of SCC25 and CAL27 cells

Subsequently, si-SNHG20 and the miR-19b-3p inhibitor have been co-transfected into SCC25 and CAL27 cells. The expression of miR-19b-3p was sharply downregulated after miR-19b-3p inhibitor transfection (Figure 4a). Furthermore, the knockdown of miR-19b-3p promoted the proliferation of SCC25 and CAL27 cells (Figure 4b). Similarly, the knockdown of miR-19b-3p upregulated the growth and metastasis of SCC25 and CAL27 cells (Figure 4c-d).

(A) RT-qPCR analysis of miR-19b-3p in SCC25 and CAL27 cells transfected with si-SNHG20 and miR-19b-3p inhibitor. (B) An MTT assay was used to explore the effect of si-SNHG20 and miR-19b-3p inhibitor on the proliferation of CAL27 and SCC25 cells. The number of migratory (C) and invasive (D) cells was determined using Transwell assays. Data are presented as mean ± SD. ** p < 0.01 vs. si-NC group; # p < 0.05 vs. si-SNHG20 + inhibitor NC group.

#### RAB14 *is the target gene of miR-19b-3p in OSCC*

**Figure 5.**
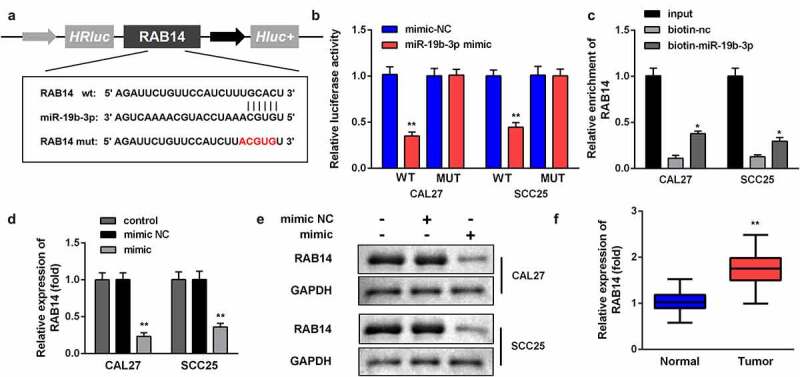
*RAB14* is the target gene of miR-19b-3p in OSCC

Furthermore, the targeting sites of miR-19b-3p and RAB14 were estimated by using the online TargetScan software (Figure 5a) and validated using a dual luciferase reporter gene system and a pull-down assay. The data showed that miR-19b-3p mimic sharply downregulated the luciferase activity in cells transfected with WT-RAB14, in place of Mut-RAB14, in SCC25 and CAL27 cells (Figure 5b). In addition, the luciferase reporter assay results were validated by the RAB14 probe pull-down assay (Figure 5c), indicating that RAB14 is a direct target of miR-19b-3p. Moreover, compared with the adjacent non-tumor tissues, RAB14 expression was dramatically increased in OSCC tissues and the overexpression of miR-19b-3p promoted the mRNA and protein expression of RAB14 (Figure 5d-f).

(A) The predicted RAB14 binding site in the miR-19b-3p 3'-UTR. (B) Relative luciferase activity of cells after co-transfection with wild type (WT) or mutant (Mut) RAB14 3'-UTR reporter genes and miR-19b-3p mimics. (C) The RNA pull-down assay further confirmed that RAB14 could bind to miR-19b-3p. RT-qPCR (D) and western blot (E) analysis were used to explore the effect of miR-19b-3p mimic on the miR-19b-3p mRNA and protein expression in CAL27 and SCC25 cells. (F) RT-qPCR analysis of RAB14 in OSCC tissues. Data are presented as mean ± SD. * p < 0.05; ** p < 0.01.

### Overexpression of RAB14 reversed the effect of miR-19b-3p mimic on the proliferation, migration, and invasion of SCC25 and CAL27 cells

**Figure 6.**
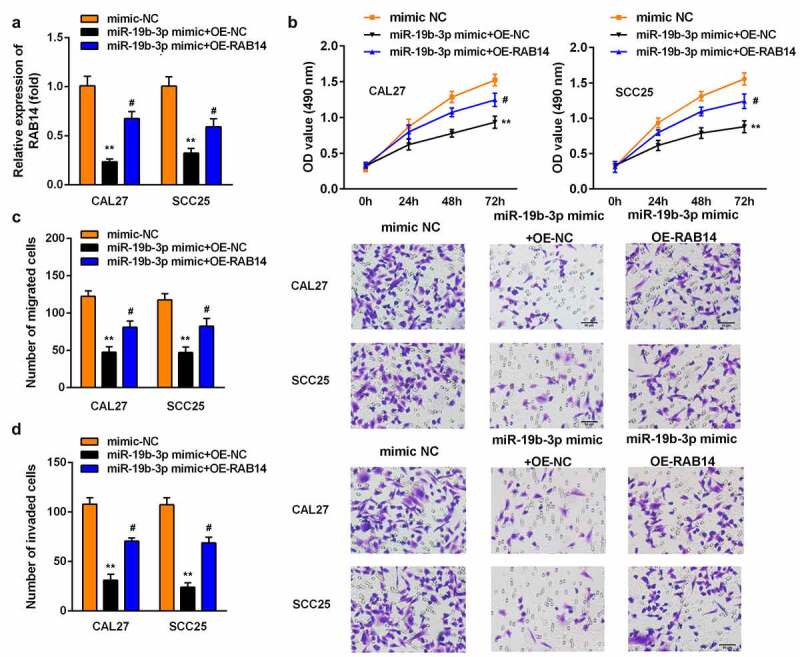
Overexpression of RAB14 reversed the effect of miR-19b-3p mimic on the proliferation, migration, and invasion of SCC25 and CAL27 cells

Last, the miR-19b-3p mimic and OE-RAB14 were transfected into SCC25 and CAL27 cells. The level of RAB14 was significantly lower after OE-RAB14 transfection (Figure 6a). The overexpression of RAB14 promoted the proliferation of SCC25 and CAL27 cells (Figure 6b). Similarly, the Transwell assay results suggested that the overexpression of RAB14 promoted the growth and metastasis of SCC25 and CAL27 cells (Figure 6c-d).

(A) RT-qPCR analysis of RAB14 in SCC25 and CAL27 cells transfected with miR-19b-3p mimic and OE-RAB14. (B) An MTT assay was used to explore the effect of miR-19b-3p mimic and OE-RAB14 on the proliferation of CAL27 and SCC25 cells. The number of migratory (C) and invasive (D) cells was determined using Transwell assays. Data are presented as mean ± SD. ** p < 0.01 vs. mimic NC group; # p < 0.05 vs. miR-19b-3p mimic and Ad-NC group.

## Discussion

In this study, we found that the expression of lncRNA SNHG20 was increased in OSCC tissues and that the knockdown of lncRNA SNHG20 could effectively suppress the biological activity of SCC25 and CAL27 cells. Our study indicated that lncRNA SNHG20 suppresses the biological activities of OSCC by regulating the miR-19b-3p/RAB14 axis.

Previous studies have shown that lncRNA can not only change the chromosome concept through the interaction of chromosome-modifying enzymes, but also silence the expression of a variety of miRNAs [23]. Therefore, lncRNA can also be used as an intervention target in tumor therapy. LncRNA SNHG20 was first isolated from the cDNA subtractive library of tumor suppressor gene by Zhang et al. and it was located on chromosome [24]. There is growing evidence indicating that lncRNA SNHG20 is closely involved in the development of many cancer processes. However, the research on lncRNA SNHG20 in OSCC is still limited. In this study, we found that, compared with the adjacent non-tumor tissues, the level of lncRNA SNHG20 was increased in OSCC tissues. In addition, we found that the knockdown of lncRNA SNHG20 can sharply inhibit the growth and metastasis of SCC25 and CAL27 cells, which indicates that lncRNA SNHG20 plays a role in promoting cancer during the progression of OSCC. Li et al. [25] also found that lncRNA SNHG20 can promote the growth of laryngeal squamous cell carcinoma cells, similar to our results.

Additionally, lncRNA can affect tumor progression by regulating miRNA expression [26,27]. In order to further analyze the functional mechanism of lncRNA SNHG20 in OSCC, here, using bioinformatics, we predicted that lncRNA SNHG20 could target and negatively regulate miR-19b-3p. miRNAs belong to a group of small noncoding RNAs that regulate gene expression at the post-transcriptional level. Previous evidence has confirmed that lncRNA SNHG20 increases the proliferation of laryngeal squamous cell carcinoma cells by regulating miR-140 [25]; Wu et al. [28] also found that lncRNA SNHG20 increases the proliferation and invasion of prostate cancer cells by targeting miR-6516-5p. In this study, we predicted that lncRNA SNHG20 has a binding site where it binds to miR-19b-3p, and the luciferase reporter pull-down assay confirmed that lncRNA SNHG20 can target and regulate miR-19b-3p. Then, we detected the level of miR-19b-3p in OSCC tissues and found that miR-19b-3p was downregulated. miR-19b-3p has been explored in various cancers, such as breast, pancreatic, and colon cancer [29–31]. MicroRNA profiling in patients with gastric cancer showed that the level of miR-19b-3p was remarkably decreased, similar to our results [32]. Furthermore, the knockdown of lncRNA SNHG20 can downregulate the level of miR-19b-3p in CAL27 and SCC25 cells. In the rescue experiment, the knockdown of miR-19b-3p reversed the effect of si-SNHG20 on the growth and metastasis of SCC25 and CAL27 cells. These results demonstrated the possible mechanism by which lncRNA SNHG20 regulates the biological behavior of OSCC cells by affecting the level of miR-19b-3p.

As reported in gastric cancer, miR-19b-3p was downregulated and negatively regulated neuropilin-1 [33]. In this study, through bioinformatics prediction and luciferase reporter and pull-down assay, *RAB14* was found to be a potential target gene of miR-19b-3p. At the same time, the level of RAB14 was increased in OSCC tissues. Rab family proteins attach an essential part in carcinogenesis and promote the malignant development of tumors by influencing cell proliferation, differentiation, apoptosis, and various cell signaling pathways [15,16]. According to previous research, the mRNA expression of RAB14 in gastric cancer is dramatically higher than that in healthy gastric mucosa; the abnormal expression of RAB14 in gastric tumors promotes the malignant development of tumors by activating the Akt signaling pathway [15]. In kidney cancer cells, *RAB14*, which is a target gene of miR-148a, promotes the growth and metastasis of tumor cells, and enhances the chemotherapy resistance of tumor cells [16]. The increased expression of RAB14 in ovarian cancer promotes tumor cell proliferation and invasion through the Wnt signaling pathway [17]. In this study, the overexpression of miR-19b-3p dramatically decreased the mRNA and protein expression of RAB14. However, the overexpression of RAB14 reversed the effect of miR-19b-3p mimic on the proliferation, migration, and invasion of SCC25 and CAL27 cells, further demonstrating that *RAB14* is a vital target gene for lncRNA SNHG20 and miR-19b-3p.

## Conclusion

In brief, our data showed that lncRNA SNHG20 is significantly upregulated in OSCC and promotes the growth and metastasis of SCC25 and CAL27 cells by absorbing miR-19b-3p to upregulate the RAB14 level. This study provided a new idea and theoretical basis for the diagnosis and treatment of OSCC.

## Data Availability

The datasets used and/or analyzed during the current study are available from the corresponding author upon reasonable request.

## Article highlights

1. SNHG20 was up-regulated in OSCC
2. SNHG20 regulated OSCC cell functions by sponging miR-19b-3p
3. RAB14 is the target gene of miR-19b-3p in OSCC

## References

[1] Morgensztern D, Campo MJ, Dahlberg SE, et al. Molecularly targeted therapies in non–small-cell lung cancer annual update 2014. J Thorac Oncol. 2015;10:S1–S63. 25535693 10.1097/JTO.0000000000000405 PMC4346098

[2] Alberg AJ, Brock MV, Ford JG, et al. Epidemiology of lung cancer: diagnosis and management of lung cancer, 3rd ed: American College of chest physicians evidence-based clinical practice guidelines. Chest. 2013;143:e1S–e29S. 23649439 10.1378/chest.12-2345 PMC4694610

[3] Shtivelman E, Hensing T, Simon GR, et al. Molecular pathways and therapeutic targets in lung cancer. Oncotarget. 2014;5(6):1392–1433. 24722523 10.18632/oncotarget.1891 PMC4039220

[4] Wu X, Wu G, Yao X, et al. The clinicopathological significance and ethnic difference of FHIT hypermethylation in non-small-cell lung carcinoma: a meta-analysis and literature review. Drug Des Devel Ther. 2016;10:699–709. 10.2147/DDDT.S85253 PMC4760666 26929601

[5] Lemjabbar-Alaoui H, Hassan OU, Yang Y, et al. Lung cancer: biology and treatment options, Biochimica et biophysica acta. Rev Cancer. 2015;1856:189–210. 10.1016/j.bbcan.2015.08.002 PMC4663145 26297204

[6] Socinski MA, Evans T, Gettinger S, et al. Treatment of stage IV non-small cell lung cancer: diagnosis and management of lung cancer, 3rd ed: American College of chest physicians evidence-based clinical practice guidelines. Chest. 2013;143(5):e341S–e368S. 23649446 10.1378/chest.12-2361 PMC4694611

[7] Farhat FS, Houhou W. Targeted therapies in non-small cell lung carcinoma: what have we achieved so far? Ther Adv Med Oncol. 2013;5(4):249–270. 23858333 10.1177/1758834013492001 PMC3707340

[8] Sun M, Kraus WL. From discovery to function: the expanding roles of long noncoding RNAs in physiology and disease. Endocr Rev. 2015;36:25–64. 25426780 10.1210/er.2014-1034 PMC4309736

[9] Marchese FP, Raimondi I, Huarte M. The multidimensional mechanisms of long noncoding RNA function. Genome Biol. 2017;18(1):206. 29084573 10.1186/s13059-017-1348-2 PMC5663108

[10] Schmitz SU, Grote P, Herrmann BG. Mechanisms of long noncoding RNA function in development and disease. Cell Mol Life Sci. 2016;73(13):2491–2509. 27007508 10.1007/s00018-016-2174-5 PMC4894931

[11] Li C, Zhou L, He J, et al. Increased long noncoding RNA SNHG20 predicts poor prognosis in colorectal cancer. BMC Cancer. 2016;16(1):655. 27543107 10.1186/s12885-016-2719-x PMC4992210

[12] Wu J, Zhao W, Wang Z, et al. Long non‐coding RNA SNHG20 promotes the tumorigenesis of oral squamous cell carcinoma via targeting miR‐197/LIN28 axis. J Cell Mol Med. 2019;23(1):680–688. 30394668 10.1111/jcmm.13987 PMC6307847

[13] Seixas E, Ramalho JS, Mota LJ, et al. Bacteria and protozoa differentially modulate the expression of Rab proteins. PLoS One. 2012;7(7):e39858. 22911692 10.1371/journal.pone.0039858 PMC3401185

[14] Lu R, Johnson DL, Stewart L, et al. Rab14 regulation of claudin-2 trafficking modulates epithelial permeability and lumen morphogenesis. Mol Biol Cell. 2014;25(11):1744–1754. 24694596 10.1091/mbc.E13-12-0724 PMC4038501

[15] Guo B, Wang W, Zhao Z, et al. RAB14 act as oncogene and induce proliferation of gastric cancer cells via AKT signaling pathway. PLoS One. 2017;12:e170620. 10.1371/journal.pone.0170620 PMC5249107 28107526

[16] Kim EA, Kim TG, Sung EG, et al. miR-148a increases the sensitivity to cisplatin by targeting RAB14 in renal cancer cells. Int J Oncol. 2017;50(3):984–992. 28098870 10.3892/ijo.2017.3851

[17] Hou R, Jiang L, Yang Z, et al. Rab14 is overexpressed in ovarian cancers and promotes ovarian cancer proliferation through Wnt pathway. Tumor Biol. 2016;37(12):16005–16013. 10.1007/s13277-016-5420-4 27718127

[18] Shang J, Sun S, Zhang L, et al. miR-211 alleviates ischaemia/reperfusion-induced kidney injury by targeting TGFβR2/TGF-β/SMAD3 pathway. Bioengineered. 2020;11(1):547–557. 32375588 10.1080/21655979.2020.1765501 PMC8291827

[19] Tang C, Feng W, Bao Y, et al. Long non-coding RNA TINCR promotes hepatocellular carcinoma proliferation and invasion via STAT3 signaling by direct interacting with T-cell protein tyrosine phosphatase (TCPTP). Bioengineered. 2021;12(1):2119–2131. 34057016 10.1080/21655979.2021.1930336 PMC8806792

[20] Liu K, Zhao D, Wang D. LINC00528 regulates myocardial infarction by targeting the miR-143-3p/COX-2 axis. Bioengineered. 2020;11(1):11–18. 31833800 10.1080/21655979.2019.1704535 PMC6961595

[21] Shang D, Liu Y, Zhang J, et al. Peroxisome proliferator-activated receptor γ (PPARγ) suppresses the proliferation and metastasis of patients with urothelial carcinoma after renal transplantation by inhibiting LEF1/β-catenin signaling. Bioengineered. 2020;11(1):1350–1367. 33289586 10.1080/21655979.2020.1843834 PMC8291807

[22] Zhang N, Liu JF. MicroRNA (MiR)-301a-3p regulates the proliferation of esophageal squamous cells via targeting PTEN. Bioengineered. 2020;11(1):972–983. 32970954 10.1080/21655979.2020.1814658 PMC8291791

[23] Chen J, Zhang K, Song H, et al. Long noncoding RNA CCAT1 acts as an oncogene and promotes chemoresistance in docetaxel-resistant lung adenocarcinoma cells. Oncotarget. 2016;7(38):62474–62489. 27566568 10.18632/oncotarget.11518 PMC5308740

[24] Zhang D, Cao C, Liu L, et al. Up-regulation of LncRNA SNHG20 predicts poor prognosis in hepatocellular carcinoma. J Cancer. 2016;7(5):608–617. 27053960 10.7150/jca.13822 PMC4820738

[25] Li Y, Xu J, Guo Y, et al. LncRNA SNHG20 promotes the development of laryngeal squamous cell carcinoma by regulating miR-140. Eur Rev Med Pharmacol Sci. 2019;23:3401–3409. 31081112 10.26355/eurrev_201904_17704

[26] Chan JJ, Tay Y. Noncoding RNA:RNA regulatory networks in cancer. Int J Mol Sci. 2018;19(5):1310. 10.3390/ijms19051310 PMC5983611 29702599

[27] Wang J, Yang Y, Ma Y, et al. Potential regulatory role of lncRNA-miRNA-mRNA axis in osteosarcoma. Biomed Pharmacother. 2020;121:109627. 31810120 10.1016/j.biopha.2019.109627

[28] Wu X, Xiao Y, Zhou Y, et al. lncRNA SNHG20 promotes prostate cancer migration and invasion via targeting the miR-6516-5p/SCGB2A1 axis. Am J Transl Res. 2019;11:5162–5169. 31497231 PMC6731428

[29] Song M, Sun M, Xia L, et al. miR-19b-3p promotes human pancreatic cancer Capan-2 cells proliferation by targeting phosphatase and tension homolog. Ann Transl Med. 2019;7(11):236. 31317006 10.21037/atm.2019.04.61 PMC6603353

[30] Jin J, Sun Z, Yang F, et al. miR-19b-3p inhibits breast cancer cell proliferation and reverses saracatinib-resistance by regulating PI3K/Akt pathway. Arch Biochem Biophys. 2018;645:54–60. 29550144 10.1016/j.abb.2018.03.015

[31] Jiang T, Ye L, Han Z, et al. miR-19b-3p promotes colon cancer proliferation and oxaliplatin-based chemoresistance by targeting SMAD4: validation by bioinformatics and experimental analyses. J Exp Clin Cancer Res. 2017;36(1):131. 28938919 10.1186/s13046-017-0602-5 PMC5610468

[32] Zhang T, Liu C, Huang S, et al. A downmodulated microRNA profiling in patients with gastric cancer. Gastroenterol Res Pract. 2017;2017:1526981–1526988. 28546810 10.1155/2017/1526981 PMC5436063

[33] Wei Y, Guo S, Tang J, et al. MicroRNA-19b-3p suppresses gastric cancer development by negatively regulating neuropilin-1. Cancer Cell Int. 2020;20(1):193. 32508529 10.1186/s12935-020-01257-0 PMC7249695

